# Increased Live Birth Rate with Dydrogesterone among Patients with Recurrent Pregnancy Loss Regardless of Other Treatments

**DOI:** 10.3390/jcm12051967

**Published:** 2023-03-02

**Authors:** Asher Bashiri, Gabi Galperin, Atif Zeadna, Yael Baumfeld, Tamar Wainstock

**Affiliations:** 1 Department of Obstetrics and Gynecology, Soroka University Medical Center, Faculty of Health Sciences, Ben-Gurion University of the Negev, Beer Sheva 84101, Israel; 2 IVF Unit, Division of Obstetrics and Gynecology, Soroka University Medical Center, Faculty of Health Sciences, Ben-Gurion University of the Negev, Beer Sheva 84101, Israel; 3 Department of Public Health, Faculty of Health Sciences, Ben-Gurion University of the Negev, Beer Sheva 84101, Israel

**Keywords:** recurrent pregnancy loss (RPL), recurrent miscarriage, progesterone, dydrogesterone, progestins, progestogens, live birth

## Abstract

Background: Recurrent pregnancy loss (RPL) is defined as the loss of two or more pregnancies. Several treatment options are available, including progesterone, which is one of the few treatments that improve live birth rates in RPL patients. Objective: To compare the live birth rates, medical and obstetric characteristics, and RPL evaluation results of women with and without progesterone treatment. These women attended the RPL clinic at Soroka University Medical Center. Methods: A retrospective cohort study based on 866 patients was conducted. The patients were divided into two groups and examined: the dydrogesterone treatment group consisting of 509 women and a group of 357 patients who did not receive the treatment. All the patients had a subsequent (index) pregnancy. Results: The two groups were not statistically different in terms of their demographic and clinical characteristics or evaluation results. In a univariate analysis, no statistically significant differences were found between the groups in terms of live birth rates (80.6% vs. 84%; *p*-value = 0.209). In a multivariate logistic analysis adjusted for maternal age, the ratio of pregnancy losses to the number of pregnancies, other administered treatments, antiphospholipid syndrome, and body mass index, dydrogesterone treatment was found to be independently associated with a higher rate of live births than the control group (adjusted OR = 1.592; CI 95% 1.051–2.413; *p*-value = 0.028). Conclusions: Progesterone treatment is associated with an increased live birth rate in RPL patients. Studies with larger sample sizes are recommended to strengthen these results.

## 1. Introduction 

According to the European Society of Human Reproduction and Embryology (ESHRE) guidelines, recurrent pregnancy loss (RPL) is defined as the loss of two or more pregnancies [1]. The American Society of Reproductive Medicine (ASRM) defines RPL as the loss of two or more clinical pregnancies [2]. Neither of these definitions requires the pregnancies to be consecutive although most women with RPL do experience a consecutive loss. 

Approximately 5% of women experience RPL [3]. According to the ESHRE guidelines, the mandatory evaluation for women with RPL includes an assessment of anatomical factors, acquired thrombophilia, uterine abnormalities and thyroid factors. A healthy lifestyle has been found to have a positive influence on live birth rates. Nonetheless, approximately 50% of RPL cases are defined as unexplained if the product of conception does not undergo karyotyping [4]. Treatment options in the unexplained group are complex as information regarding the efficacy of treatments is only partial. Among the treatments explored in the literature, there are anticoagulation, immunological, psychological, and progesterone options. Progesterone treatment is the only one found to be effective in improving the live birth rate according to evidence-based medicine [5].

Progesterone, a steroid hormone, creates a suitable endometrial environment for implantation [6]. Hence, it is essential for the achievement and maintenance of pregnancy. It is impossible to measure progesterone levels in pregnancy because of its pulsatile secretion [7]. Several progesterone drugs exist, and they are different in terms of structure as well as in terms of their pharmacokinetics and affinities for binding with the different steroid receptors specific to progesterone [8].

Several studies have found positive effects of dydrogesterone treatment in RPL patients. A randomized controlled trial conducted by El Zibdeh et al. [9] reported that in a group of women who received dydrogesterone treatment, the chances of spontaneous abortion were reduced compared to women in the control group, who received no additional treatment (13.4% vs. 29%, respectively; *p*-value ≤ 0.05). In another randomized controlled trial conducted by Kumar et al. [10,11], it was found that the administration of dydrogesterone in early pregnancy improved pregnancy outcomes in women with three or more unexplained pregnancy losses (16.8% pregnancy losses in the control group vs. 6.9% in the treatment group; *p*-value = 0.04). Moreover, the mean gestational age at birth was significantly higher in the treatment group than in the control group (38.01 ± 2.0 weeks vs. 37.23 ± 2.4, respectively; *p*-value = 0.02).

Several meta-analyses have shown the effectiveness of progestative treatment in women with unexplained RPL. Among these is a study by Saccone et al. [12], who compared supplementation with a variety of progestogens, including natural P and synthetic progestins, and found that progestogens were associated with a reduced incidence of recurrent miscarriages (RR = 0.72; 95% CI 0.53–0.97). Of these progestogens, dydrogesterone was found to be the most effective.

A Cochrane review by Haas et al. [13] examined synthetic progesterone and natural progesterone and found that there was a reduced rate of pregnancy losses after treatment with progesterone (OR = 0.39; 95% CI 0.21–0.72) in women with three or more pregnancy losses. This difference was not significant in women with two pregnancy losses. 

Conversely, Coomarasamy et al. [14] found no significant difference in the live birth rate in women with RPL who received micronized vaginal progesterone (MVP) in the first trimester versus the placebo group, while the patients in the placebo group had three or more pregnancy losses (65.8% in the treatment group vs. 63.3%; RR = 1.04; 95% CI 0.94–1.15; *p*-value = 0.45).

Considering the inconsistent findings on RPL treatment and the fact that in women who experience RPL—especially those in the “unexplained” group—a component of insufficient progesterone level or abnormalities in the progesterone receptor, affinity, or all of these, may increase the pregnancy loss rate, we sought to determine whether there was an association between progesterone treatment—specifically with dydrogesterone—of women with RPL and live birth rate. In addition, there may be immunological imbalances in these patients, and their pregnancy outcomes may be improved by the immunomodulation of dydrogesterone [15].

## 2. Materials and Methods

### 2.1. Study Population

A retrospective cohort study based on women who attended the Soroka University Medical Center (SUMC) RPL clinic between 2016 and 2022 was conducted. The women were divided into two groups: the study group, which received dydrogesterone, and the control group, which did not. An index pregnancy was defined as the first documented pregnancy following an investigation at the clinic.

The progesterone administered was an oral administration of dydrogestrone 10 mg twice daily. The recommended use was from pregnancy diagnosis until 20 weeks’ gestation.

SUMC is a tertiary center and the sole medical facility in the southern region of Israel. The RPL clinic accepts referred patients who have been diagnosed with RPL. At the clinic, women are examined, undergo an extensive evaluation, and receive consultation and treatment in accordance with RPL etiology and risk factors and in conformity with the latest guidelines as well as regular follow-ups during the index pregnancy and later pregnancies. All women included in this study had experienced two or more pregnancy losses. 

Inclusion criteria: the study population included all patients admitted to the recurrent pregnancy loss clinic (RPL clinic). The study population consisted of the patients who adhered to the treatment while the control group was composed of those who did not take the medication. Evaluation of treatment was performed using computerized medical records which include the record of medications bought. 

### 2.2. Data Collection

All participating women underwent partial or complete RPL evaluation during their first visit to the RPL clinic. Thorough medical and obstetric histories were taken, and physical examinations were performed. Data regarding the results of previous pregnancy loss evaluations were recorded. The following demographic and clinical information was obtained: age, occupation, ethnicity, consanguinity, chronic diseases, routine medication use, and obstetric history. Results of the RPL evaluation were recorded during follow-up sessions. During pregnancy, patients were seen at the RPL clinic every 3 to 8 weeks until delivery depending on all the clinical parameters. 

All data collected for the study, including drug prescription documentation, were drawn from SUMC’s information technology unit and stored in a Microsoft Access database.

## 3. Clinical Laboratory Investigation and Tests

Patients were evaluated and underwent further investigation in accordance with their history, examination, and an expert physician’s discretion. The conditions assessed included the following:

### 3.1. Uterine Anatomic Defects

Anatomic evaluation of the reproductive tract was performed using hysteroscopy or 3-D ultrasound. Abnormal findings included congenital uterine anomalies, fibroids, polyps >1.0 cm in size, and Asherman’s syndrome. A uterine anomaly was defined as one or more abnormal findings.

### 3.2. Endocrine Abnormalities

Endocrine evaluation included thyroid function (serum TSH level > 4 mIU/L was considered abnormal), antithyroid peroxidase level (>40 was considered abnormal), antithyroid antibody level (>35 was considered abnormal), serum fasting glucose level (>126 mg/dL was considered abnormal), HbA1C (HbA1C > 5.7% was considered abnormal), and serum prolactin level (>29 ng/mL was considered abnormal).

### 3.3. Autoimmune Abnormalities

Anticardiolipin (ACL) antibodies—Serum levels of ACL IgG and IgM were measured using enzyme-linked immunoassay. Levels exceeding 18 U/mL were considered abnormal. All positive tests were confirmed by repeated testing at least 12 weeks later.

Anti-β2-glycoprotein-1 antibodies—Serum levels of anti-β2-glycoprotein-1 IgM and IgG were measured using enzyme-linked immunoassay. Levels exceeding 18 U/mL were considered abnormal.

Lupus anticoagulant (LAC)—was considered abnormal when RVVT-LAC ratio was above 1.3 and/or SCT-LAC ratio was above 1.2.

### 3.4. Hereditary Thrombophilia 

A workup included the following: serum levels of functional protein C activity (levels < 69% were considered abnormal), serum levels of functional protein S activity (levels < 57.6% were considered abnormal), antithrombin activity (levels < 59.1% were considered abnormal), prothrombin gene mutation (heterozygous or homozygous mutations of the G20210A prothrombin gene), and factor V Leiden mutation (considered abnormal if a heterozygous or homozygous factor V Leiden G1691A mutation was found). Inherited thrombophilia was defined as the presence of any abnormalities in one or more test results.

### 3.5. Parental Chromosomal Abnormalities

Chromosome studies were performed on cultured lymphocytes. Slides were processed for G-banding using standard techniques employing trypsin–Giemsa. Colchicine was added 4 h before the cytological samples were prepared. For the karyotype analysis, a minimum of 15 cells in metaphase from 2 independent cultures were microscopically analyzed. Chromosomal aberrations included chromosomal inversions, microdeletions, and mainly balanced translocations, such as reciprocal and Robertsonian translocations. Abnormalities in either or both parents were considered a positive finding.

## 4. Results

In the 6 years under study, 1474 women attended the RPL clinic and underwent partial or complete evaluation. Of these, 866 patients had an index pregnancy and were included in the study. The remaining 608 patients were excluded due to a lack of data on the index pregnancy. 

Table 1 presents a comparison of the demographic, clinical, and index pregnancy characteristics of the study population. No significant differences were found between the intervention and control groups regarding maternal age, number of previous pregnancy losses, consanguineous marriages, body mass index (BMI), lifestyle habits (including smoking and alcohol consumption), or chronic diseases. Women who received progesterone were more likely to be diagnosed with antiphospholipid syndrome (APS) than the control group (18.5% vs. 10.1%, respectively; *p*-value = 0.001). 

Table 2 presents a comparison of the evaluation tests between the groups. There were no statistically significant differences between the groups in any of the evaluation tests with the exception of A.B2 glycoprotein IgM levels, which were higher in the control group than in the dydrogesterone -treated group (1.8% vs. 6.9%; *p*-value = 0.032).

Table 3 presents the index pregnancy results, including the primary outcome of live births, which were not statistically different between the study and control groups (80.6% vs. 84.0%, respectively; *p*-value = 0.209). There were no differences between the groups in terms of mean maternal age at index pregnancy. Women in the study group received other treatments in addition to dydrogesterone, including vitamin D (7.9% vs. 1.7%; *p*-value < 0.001), Eltroxin (11.2% vs. 6.7%; *p*-value = 0.032), Clexane (34.6% vs. 24.4%; *p*-value = 0.001), Prednisone (12.6% vs. 4.2%; *p*-value < 0.001), and Aspirin (15.5% vs. 7.3%; *p*-value < 0.001).

The multivariate logistic analysis adjusted for maternal age at index pregnancy, the ratio of pregnancy losses to the number of pregnancies, any other treatments, APS, and BMI is presented in Table 4. Dydrogesterone was found to have an independent and statistically significant positive association with live births (adjusted OR = 1.592; CI 95% 1.051–2.413; *p*-value = 0.028). 

Table A1 in Appendix A shows the demographic and clinical characteristics of all 1474 women who attended the RPL clinic and received dydrogesterone treatment versus those who received no dydrogesterone treatment.

## 5. Discussion

### 5.1. Principal Findings

In this study, the impact of dydrogesterone treatment on pregnancy outcomes was evaluated among patients with RPL. Although the live birth rate was not statistically higher in the group of women treated with dydrogesterone, after adjusting for the main confounding and clinically relevant variables, the dydrogesterone treatment was associated with a higher live birth rate. 

### 5.2. Results

Both groups’ live birth rates are considered good for women with RPL [6]. At the RPL clinic, patients are closely monitored, have immediate access to treatment, and are treated with tender, loving care [4] in addition to receiving specific treatments. All of these contribute to good live birth rates. We consider the clinic a significant factor in generally improving outcomes.

In order to determine whether dydrogesterone treatment is an independent factor in live birth among women with RPL, we performed several multivariate analyses. The model in Table 4 and all other analyses performed showed that dydrogesterone has an independent and statistically significant positive effect on the live birth rate (OR = 1.592; CI 95% for OR 1.051–2.413; *p* = 0.028).

Our findings are supported by several papers, including meta-analyses and systematic reviews. Kumar et al. [10] reported that the administration of dydrogesterone in early pregnancy can improve pregnancy outcomes in women with three or more unexplained pregnancy losses. Saccone et al. [12] found that dydrogesterone treatment was superior among progestogens in reducing the incidence of recurrent miscarriages. A Cochrane review by Haas et al. [13] found that there was a reduced rate of pregnancy loss after treatment with progesterone in women with three or more pregnancy losses. Nevertheless, Coomarasamy et al. [14] found no significant difference between the rates of live birth among women with RPL who received micronized progesterone therapy (Utrogestan) in the first trimester and the women in a placebo group.

One might consider the progesterone preparation to affect bioavailability and pregnancy outcome. In our study, we explore dydrogesterone, which is known for its improved bioavailability and metabolic stability. This could explain the differences between the different studies and the positive effect of the treatment we found in our study.

Several mechanisms may explain the positive effect of progesterone treatment in RPL patients. One of the most important explanations is progesterone’s immunomodulatory effect [15]. Another possible mechanism is the presence of an abnormality in progesterone levels, affinity, or both. 

The usage of additional treatments (Vitamin D, Levothyroxine, Enoxaparin, Prednisone, and Aspirin) was higher in the study group than in the control group. This could be due to patient compliance, assuming that patients who were already given certain treatments had more complex conditions and therefore were more willing to be treated with progesterone and adhered better to the treatment than the patients in the control group. It is important to emphasize that none of the additional treatments changed the positive effect of progesterone. 

We adjusted for these additional interventions in the multivariate analysis, and our findings were independently statistically significant.

In our analysis, advanced maternal age was clearly associated with low live birth rates and high ratio of pregnancy losses to the number of pregnancies. These findings are well known and supported by literature [16]. Therefore, it is of utmost importance to conduct evaluations as early as possible when women meet RPL criteria so that the probability of live birth will not be reduced by their advanced age. 

The RPL evaluation results in our study were similar to those reported in the literature [17,18]. There were no statistically significant differences between the groups. 

### 5.3. Strengths and Limitations

Our study has several limitations. First, it is a retrospective study, and as such no conclusions regrading causality can be made. Secondly, adherence to the treatment could not be precisely evaluated. Moreover, approximately 608 women did not have index pregnancies, and this may be due to the short time that passed from the time of the evaluation to the end of the study period. In addition, although SUMC is a tertiary medical center that serves the entire population of southern Israel, we have no data regarding women who prefer to deliver in other hospitals, and therefore data may be missing. 

Nonetheless, our study has several important strengths—the main one being the non-selective nature of the population and the fact that SUMC is the only tertiary medical center that serves the entire population of southern Israel. Its RPL clinic is the sole such facility in the southern region; and since SUMC provides medical care to the population of this region, women who reside there are highly likely to be treated at SUMC and give birth there. Moreover, the fact that the data are based on computerized documentation makes them extremely reliable and accurate in terms of laboratory tests, dates, and patients’ demographic information and treatment documentation. 

## 6. Conclusions

An increased live birth rate was found in women treated with dydrogesterone regardless of other treatments. Studies with larger sample sizes than ours should be conducted to strengthen our conclusions. 

## Figures and Tables

**Table 1:** Demographic and clinical characteristics of women with RPL who had index pregnancies and who received dydrogesterone treatment vs. those who received no treatment.

	Dydrogesterone Treatment	No Dydrogesterone Treatment	*p*-Value
	*n* = 509	*n* = 357	
Maternal age			
Mean ± SD	29.97 ± 5.894	29.86 ± 5.994	0.8
Over 35	108 (21.2)	67 (18.8)	0.391
Number of previous pregnancy losses (mean ± SD) *	3.124 ± 1.620	3.197 ± 1.480	0.554
Consanguineous marriages	134 (26.3)	76 (21.3)	0.091
BMI *			0.154
Lower than 20	55 (12.4)	18 (8.2)	
20–24.9	178 (40.2)	81 (36.8)	
25–29.9	108 (24.4)	68 (38.6)	
Higher than 29.9	102 (23.0)	53 (24.1)	
BMI ≥ 25	210 (47.4)	121 (55.0)	0.07
Smoking *	34 (6.8)	16 (6.6)	1
Alcohol use *	0 (-)	0 (-)	-
Spontaneous pregnancy	381 (87.8)	285 (91.6)	0.116
Chronic diseases			
APS	94 (18.5)	36 (10.1)	0.001
Hypothyroidism	41 (8.1)	42 (11.8)	0.078
Hypertension	10 (2.0)	5 (1.4)	0.606
Diabetes	15 (2.9)	15 (4.2)	0.349

* Missing values: number of previous pregnancy losses: *n* = 118; BMI: *n* = 203; smoking: *n* = 121; alcohol use: *n* = 121; spontaneous pregnancy: *n* = 121.

**Table 2:** RPL Evaluation of abnormal results among women with index pregnancies.

	Dydrogesterone Treatment *n* = 509 *	No Dydrogesterone Treatment *n* = 357 *	*p*-Value
Anatomical evaluation (Abnormal/Tested)	4.3 (6/141)	7.4 (2/27)	0.616
Karyotype (Abnormal/Tested)	- (0/39)	- (0/11)	-
TSH (Abnormal/Tested)	10.3 (48/466)	6.1 (17/279)	0.063
Prolactin (Abnormal/Tested)	11.1 (26/234)	8.9 (8/90)	0.687
Glucose (Abnormal/Tested)	34.4 (33/96)	31.8 (7/22)	1.000
HbA1C (Abnormal/Tested)	6.9 (2/29)	- (0/4)	1.000
Anti-thyroid peroxidase (Abnormal/Tested)	14.1 (26/185)	9.7 (6/62)	0.513
Antithyroglobulin (Abnormal/Tested)	7.8 (13/166)	12.1 (7/58)	0.421
Lupus anticoagulant (LAC)	2.3 (6/265)	1.6 (1/63)	1.000
ACL IgG (Abnormal/Tested)	2.8 (7/250)	1.0 (1/99)	0.449
ACL IgM (Abnormal/Tested)	2.0 (5/255)	1.0 (1/101)	1.000
A.B2 glycoprotein IgG (Abnormal/Tested)	0.5 (1/219)	1.2 (1/85)	0.482
A.B2 glycoprotein IgM (Abnormal/Tested)	1.8 (4/224)	6.9 (6/87)	0.032
Antinuclear Ab (Abnormal/Tested)	10.2 (17/167)	8.1 (6/74)	0.813
Rheumatoid factor (Abnormal/Tested)	5.5 (11/200)	8.4 (7/83)	0.423
Protein C (Abnormal/Tested)	2.9 (5/170)	3.1 (2/65)	1.000
Protein S (Abnormal/Tested)	22.2 (6/27)	- (0/11)	0.154
Antithrombin 3 (Abnormal/Tested)	0.6 (1/165)	1.6 (1/64)	0.482
Factor V Leiden mutation (Abnormal/Tested)	9.1 (3/33)	37.5 (3/8)	0.048
Prothrombin mutation (Abnormal/Tested)	3.3 (1/30)	- (0/7)	1.000

* Missing values: anatomical evaluation: *n* = 698; karyotype: *n* = 816; TSH: *n* = 121; prolactin: *n* = 542; glucose: *n* = 748; HbA1C: *n* = 833; anti-thyroid peroxidase: *n* = 619; antithyroglobulin: *n* = 642; LAC: *n* = 538; ACL IgG: *n* = 517; ACL IgM: *n* = 510; A.B2 glycoprotein IgG: *n* = 562; A.B2 glycoprotein IgM: *n* = 555; antinuclear Ab: *n* = 625; rheumatoid factor: *n* = 583; protein C: *n* = 631; protein S: *n* = 828; antithrombin 3: *n* = 637; factor V Leiden mutation: *n* = 825; mutation prothrombin: *n* = 829.

**Table 3:** Index pregnancy additional treatments and results.

	Dydrogesterone Treatment *n* = 509	No Dydrogesterone Treatment *n* = 357	Adjusted Odds Ratio: 95% CI	*p*-Value
Live birth	410 (80.6)	300 (84.0)	0.787	0.209
Maternal age at index pregnancy (mean ± SD)	31.055 ± 6.040	30.484 ± 1.623		0.172
Additional treatments				
Vitamin D	40 (7.9)	6 (1.7)		0.000
Levothyroxine	57 (11.2)	24 (6.7)		0.032
Enoxaparin	176 (34.6)	87 (24.4)		0.001
Prednisone	64 (12.6)	15 (4.2)		0.000
Aspirin	79 (15.5)	26 (7.3)		0.000

**Table 4:** Multivariable analysis for the association between dydrogesterone treatment and live birth.

Variable	Adjusted OR	95% CI	*p*-Value
Dydrogesterone treatment	1.592	1.051–2.413	0.028
Advanced maternal age at index pregnancy	0.933	0.933–1.001	0.058
High ratio of pregnancy losses to number of pregnancies	0.127	0.048–0.335	0.000
Any other treatment	1.761	1.151–2.694	0.009
APS	0.440	0.271–0.713	0.001
BMI ≥ 25	0.866	0.581–1.289	0.478

## Data Availability

Data will be made available by request and depending on IRB approval.

## Appendix A

**Table A1:** Demographic and clinical characteristics of women with RPL with dydrogesterone treatment vs. those under no treatment.

	Dydrogesterone Treatment	No Dydrogesterone Treatment	*p*-Value
	*n* = 784	*n* = 690	
Maternal age			
Mean ± SD	30.80 ± 5.937	31.28 ± 6.491	0.139
Over 35	189 (24.1)	185 (26.8)	0.254
Number of previous pregnancy losses (mean ± SD) *	3.115 ± 1.623	3.169 ± 1.639	0.552
Consanguineous marriages	167 (21.3)	129 (18.7)	0.217
BMI *			0.013
Lower than 20	85 (12.2)	40 (8.0)	
20–24.9	274 (39.5)	175 (35.2)	
25–29.9	168 (24.2)	149 (30.0)	
Higher than 29.9	167 (24.1)	133 (26.8)	
BMI ≥ 25	335 (48.3)	282 (56.7)	0.004
Smoking *	60 (7.8)	43 (7.8)	1
Alcohol use *	0 (0.0)	1 (0.2)	0.415
Spontaneous pregnancy	381 (87.8)	285 (91.6)	0.116
Chronic diseases			
Hypothyroidism	44 (5.6)	45 (6.5)	0.511
Hypertension	11 (1.4)	5 (0.7)	0.314
APS	98 (12.5)	37 (5.4)	<0.001
Diabetes	16 (2.0)	16 (2.3)	0.724

* Missing values: number of previous pregnancy losses: *n* = 152; BMI: *n* = 283; smoking: *n* = 150; alcohol use: *n* = 150; spontaneous pregnancy: *n* = 729.
